# Iterative Development of an Online Dietary Recall Tool: INTAKE24

**DOI:** 10.3390/nu9020118

**Published:** 2017-02-09

**Authors:** Emma Simpson, Jennifer Bradley, Ivan Poliakov, Dan Jackson, Patrick Olivier, Ashley J. Adamson, Emma Foster

**Affiliations:** 1 Human Nutrition Research Centre, Institute of Health and Society, Newcastle University, Newcastle upon Tyne NE2 4HH, UK; Emma.simpson@ncl.ac.uk (E.S.); Jen.bradley@ncl.ac.uk (J.B.); Ashley.adamson@ncl.ac.uk (A.J.A.); 2 Digital Interaction Group, School of Computing Science, Newcastle University, Newcastle upon Tyne NE1 8HW, UK; Ivan.poliakov@ncl.ac.uk (I.P.); Dan.Jackson@ncl.ac.uk (D.J.); Patrick.olivier@ncl.ac.uk (P.O.)

**Keywords:** dietary assessment, INTAKE24, online dietary recall, web-based dietary recall, 24-h dietary recall, online dietary assessment, web-based dietary assessment

## Abstract

Collecting large-scale population data on dietary intake is challenging, particularly when resources and funding are constrained. Technology offers the potential to develop novel ways of collecting large amounts of dietary information while making it easier, more convenient, intuitive, and engaging for users. INTAKE24 is an online multiple pass 24 h dietary recall tool developed for use in national food and nutrition surveys. The development of INTAKE24 was a four-stage iterative process of user interaction and evaluation with the intended end users, 11–24 years old. A total of 80 11–24 years old took part in the evaluation, 20 at each stage. Several methods were used to elicit feedback from the users including, ‘think aloud’, ‘eye tracking’, semi-structured interviews, and a system usability scale. Each participant completed an interviewer led recall post system completion. Key system developments generated from the user feedback included a ‘flat’ interface, which uses only a single interface screen shared between all of the various activities (e.g., free text entry, looking up foods in the database, portion size estimation). Improvements to the text entry, search functionality, and navigation around the system were also influenced through feedback from users at each stage. The time to complete a recall using INTAKE24 almost halved from the initial prototype to the end system, while the agreement with an interviewer led recall improved. Further developments include testing the use of INTAKE24 with older adults and translation into other languages for international use. Our future aim is to validate the system with recovery biomarkers.

## 1. Background

Designing and developing accurate web-based dietary assessment systems for collecting large-scale population data is challenging. Dietary intake is a very complex behaviour to accurately measure and presents challenges to researchers, especially when funding and resources are constrained. Technology offers the potential to develop novel ways of collecting dietary information from large cohorts simultaneously, enabling the immediate generation of nutritional output and removing the need for manual coding and data entry typically seen with traditional dietary assessment methodology [1], eliciting a significant reduction in the cost and burden to researchers working in the field. Recently, there has been an increase in the number of web and computer based dietary assessment systems available worldwide [2,3,4,5,6,7,8]; however, few have reported on the systematic development of these systems, particularly with regards to user experience (UX) and evaluation. User evaluation is a fundamental part of the design and development process. It is important to receive feedback, particularly from the intended end users [9]. 

INTAKE24 is a self-completed online 24-h dietary recall. Log in details for the system can be emailed to study participants so they can complete the recall remotely and submit it for the researcher to access. INTAKE24 was iteratively developed from an original system, SCRAN24 [10]. SCRAN24 was a prototype developed in 9 months on a limited budget. It was based on a previous system known as IPSAS (Interactive Portion Size Assessment Software), which is the UKs only validated computer based tool used to assess the portion size of foods consumed by children and adolescents [11]. The foods and portion sizes depicted in the tool were based on the foods and portion sizes recorded by children taking part in the National Diet and Nutrition Surveys (NDNS) carried out in Great Britain [12]. SCRAN24 provided the basis of a good 24-h dietary recall system, and feedback from both students and teachers who had used the system was positive [10]. However, a number of key system developments were needed to improve the usability of the system and overall accuracy, which led to the development of INTAKE24.

INTAKE24 uses the multiple pass recall method, a process whereby the user recalls everything consumed over the previous 24 h (from waking up to when they go to sleep). The user firstly lists all food and drinks consumed, followed by probing questions about quantities and further information on the foods and drinks reported. Finally, the user is able to review all foods and drinks inputted and this provides a further opportunity to recall any missed items or information. The aim of the system is to act as the ‘interviewer’, probing for additional information, which would typically be asked during an interviewer-led recall. 

The development of INTAKE24 was a collaborative project with Open Lab and the Human Nutrition Research Centre at Newcastle University. This paper describes the iterative design process that led to the development of INTAKE24 and proposes future developments.

## 2. System Development

### 2.1. Methodology

The system was developed and tested using an iterative process of four cycles of user testing; the first round used the initial SCRAN24 system and subsequent rounds used prototypes developed based on the feedback received from users. Specific techniques were used to maximise feedback from intended users, which are well established in the technology development community, i.e., think aloud, eye tracking/screen grab software, and an industry standard System Usability Scale (SUS), a set of 10 questions on the user experience [13]. Interviewer-led 24 h recalls were conducted at each round of user testing following completion of the online recall. These helped to identify food and drink items forgotten during the online recall but that the researcher could elicit during the interview and informed the development of targeted prompts. Table 1 is a brief overview of the major alterations made after each round of user testing. Throughout development, missing foods and drinks and portion size photographs were added to the database at regular intervals. When alterations were made between each user testing, we shared the system with colleagues for a sense check of functionalities and to further identify a comprehensive food and drink list. 

### 2.2. User Testing

#### 2.2.1. Recruitment

Ethical approval for the study was granted by the Newcastle University Faculty of Medical Sciences Ethics Committee (00706/2013). Written consent was obtained for all participants.

A total of eighty participants were recruited into the four stage development phase; 10 participants aged 11–16 years and 10 aged 17–24 years at each stage, recruited from a local secondary school and university (students and staff) respectively. The 11–16 years old were recruited through a local secondary school via the food technology department. A letter outlining the study was sent to the children and their parents/carers. This requested consent, via the opt-in method, for participating in the usability testing within school food technology lessons or at another time during the school day. 

The 16–24 years old were recruited via mass emails to Newcastle University staff and students. To try and improve the representativeness of the sample, posters were displayed, with consent, at local leisure services, shopping malls, and the city library containing information about the study and contact details for those who were interested in taking part. Leaflets were also distributed in the hospital cafeteria. Although measures were put in place to reduce recruitment bias, as this is a sample of volunteers, recruitment bias is always a concern.

There were no inclusion/exclusion criteria except for the age of the participant. Usability testing and evaluation of the system was carried out on the university premises or at the secondary school, depending on the participants. Each participant was given a £10 high street shopping voucher as a token of appreciation for taking part. 

#### 2.2.2. Think Aloud

During completion of the online recall, the participants were encouraged to ‘think aloud’ [14], giving a running commentary whilst using the program. They were encouraged to say what they thought the system wanted them to do at each different stage and to comment on what worked well within the system and what didn’t. Two researchers were present at all times to observe the participants, take notes, and re-establish the flow of dialogue when the participant lapsed into silence.

#### 2.2.3. Eye Tracking

Eye tracking is the process of measuring the point of gaze (where one is looking). Eye tracking reveals which parts of the on-screen image attract the eye and the order in which the user tries to locate the relevant user interface objects.

The method used in this development study was a non-intrusive optical method; infrared light reflected from the eye is sensed by a specially designed optical sensor. This information is then analysed to extract eye rotation from changes in the reflections. A Tobii ×50 eye tracker (Tobii, Danderyd, Sweden) was used [15], an unobtrusive unit which is placed in front of the computer monitor and requires a simple calibration.

The eye movements around the screen can often help identify areas of the system interface that are confusing for the participant. For instance, repeated eye movements from one user interface object to another (such as re-reading labels or instructions multiple times) can indicate that the user is confused and unsure as to what they are expected to do next; rapid ‘panicked’ movements all over the screen indicate that the user is completely lost and cannot locate the relevant user interface elements. Unfortunately, the eye tracker software was not compatible with the school computers or the study laptops (used in the school settings). Therefore, to be able to conduct a similar type of analysis with the 11–16 years old users, a screen capture program called SnagIt was used, which captured the movement of the mouse around the screen. As the researchers made observational notes, they could detect which sections of the online recall caused the participants to stall in their progression and ask the participants to elaborate on the difficulties once the recall was complete. 

## 3. Interview and System Usability Scale

At each round of user testing, users were asked a series of questions following completion of the online system. All users were asked ‘How did you find the system overall?’ and ‘Are there parts you liked/disliked in particular?’ The remaining questions were bespoke questions developed by the researchers through observation and think aloud activity. The users were asked to provide more detail and possible suggestions for improvement on those tasks they found burdensome and comment on aspects of the system they liked or disliked. The interviews also included an industry standard System Usability Scale (SUS) [13] questionnaire in which the users rated 10 separate statements relating to the system on a scale of 1–5, strongly disagree to strongly agree, respectively; for example, ‘I think I would need help using this system’. There was also space for further comments.

## 4. Key Features

Several key features have been incorporated into INTAKE24 to improve the accuracy of dietary recalls based on user feedback at each stage of testing. The main objective was to reduce the likelihood of foods and drinks being forgotten and to make the system easy, engaging, and intuitive to use based on user feedback.

### 4.1. User Interface Design

One of the recurrent problems with the first prototype was the inconsistency in the design of different stages in the survey; each stage of the multiple pass lead to a completely different screen, with different interactions and sharing very few user interface (UI) elements with other parts of the system. Therefore, the user interface of INTAKE24 has undergone a number of significant changes from the initial prototype. Some of these changes had to be introduced in order to make the new system compatible with modern web technologies; the prototype system was designed as a stand-alone desktop application, and the presentation and user interface technologies available in a desktop environment are vastly different from those available in a web browser. INTAKE24 has also been optimized for use on tablet and mobile devices.

Most of the changes made to the aesthetics of the user interface were directly influenced by the user feedback after the first round of testing and observations gathered during the usability testing. A number of key issues arose when observing how a user navigated the system. For example, users expressed confusion when each part of the recall navigated to a new landing page, this resulted in a ‘flat interface’ being introduced, which uses only a single interface screen shared between all of the various recall activities (e.g., free text entry, looking up foods in the database, portion size estimation). 

**Figure 1:**
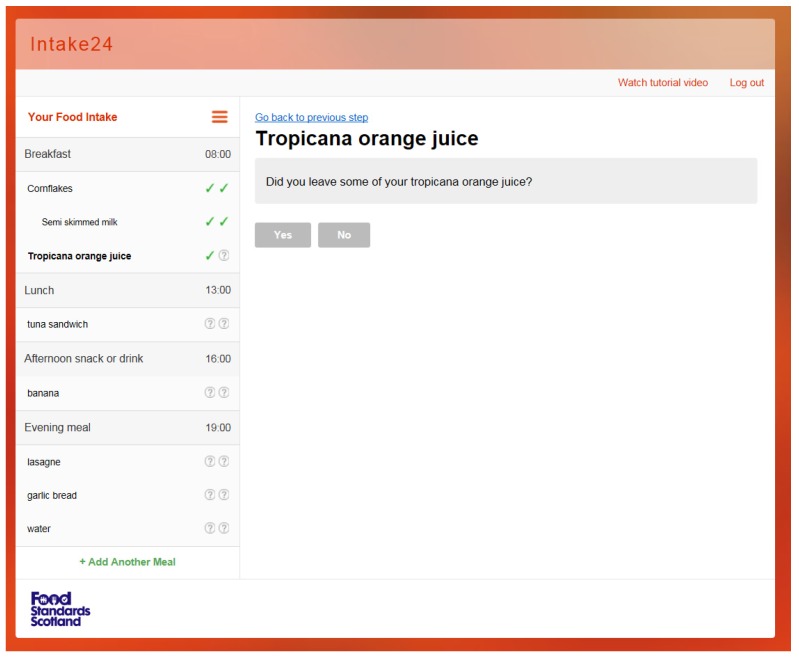
INTAKE24, contextual prompt in prompt panel. Food and drink items input shown on navigation panel.

**Figure 2:**
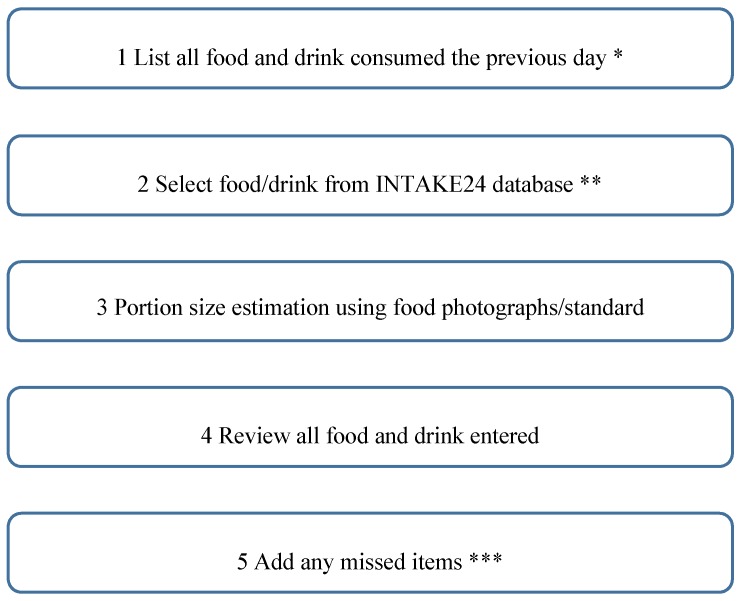
Multiple pass recall method in INTAKE24. * From when user wakes up to when they go to sleep. ** Any missing items are manually entered into the missing food function or the recipe builder. *** Associated food prompts are used to remind users of forgotten items.

As shown in Figure 1, the left hand side (called the navigation panel) always shows the current state of the survey; that is the list of meals and foods currently entered by the user and whether the user has looked up a particular food in the database and completed the portion size estimation. This makes it easy to see the overall progress and also allows the user to focus on any element (a meal or a food) at any time by clicking to expand and navigate. The list of meals is set at the start of the recall, as in Figure 2. The rationale for having a pre-defined meal list was to give structure to the recall and facilitate users in remembering the foods and drinks they consumed throughout the day. Our usability studies indicated that young people preferred the pre-defined meal list rather than an unstructured or simple ‘time of day’ style structure and were more likely to remember what they consumed. Users are able to remove the pre-defined meals; for example, selecting ‘I did not have breakfast;, would remove breakfast from the list. Users can add more meals if they require and select a meal name from a drop-down list (i.e., snack or drink, lunch) or type in their own meal name. 

The right-hand side of the interface (called the prompt panel) always shows a contextual prompt, a simple question or a short explanation of the current activity with a corresponding user interface panel; see Figure 2. When the user has answered the current question, the system will show the next prompt relevant to the currently selected item. If there are no more questions regarding the current selection, the system will automatically move to a different food or meal. The user can manually focus on an element by using the left-hand side navigation panel of the interface at any time.

The introduction of the flat interface resulted in the average time taken to complete the online recall being reduced by almost half from 22 min to 13 min across the four stages of development and the agreement with the interviewer led recall improved (see Section 6). Further, improvements made to the whole system have contributed to the decrease in completion time over the four stages of testing. 

### 4.2. Associated Food Prompts

Prompts have been included in the system, which mimic the type of questions that would be asked by a researcher in an interviewer-led recall. It is common for some foods to be forgotten; therefore associated foods have been linked to foods within the INTAKE24 system. Associated food prompts were initially based on common prompts used in interviewer-led recalls, such as butter/margarine on bread, ketchup/sauce on chips, sugar in tea/coffee etc. During user testing, we could identify commonly forgotten items when comparing the INTAKE24 recalls with interviewer-led recalls.

In addition to prompts for associated foods, if the user has not entered any drinks together with a meal, the system will ask if they are sure that they have not had any drinks with that meal. They are then given the opportunity to add these or continue with the recall if they didn’t consume any drinks. The system also recognises large gaps in time where no items have been entered, or when there has been a large calorie deficit over the day’s intake (<500 kcal); e.g., ‘Are you sure you didn’t eat or drink anything between Lunch (13:00) and Dinner (18:00)?’. There is the option to add questions to the end of the recall, probing users for further information regarding their recall, for example, ‘Did you eat as much yesterday as you would normally?’, ‘Do you follow a special diet?’, or ‘Are you taking any supplements?’.

### 4.3. Sandwich Wizard and Salad Wizard

Based on our usability testing, we identified that sandwiches and salads were commonly consumed in the age group 11–24 years. We also found that inputting these items manually often resulted in omitted items; for example, if a user input ‘tuna sandwich’ during the first pass, then the user often selected ‘tuna’ in the second pass and failed to realise that bread was missing. Due to the vast number of different sandwich fillings and combinations, it was not feasible to include every variation of sandwich within the system and keep it up to date. Therefore, a ‘build my sandwich’ wizard was created. We then iteratively developed a guide that would walk the user through each component of a sandwich or salad to reduce the risk of omissions. We implemented this after the second round of user testing. 

**Figure 3:**
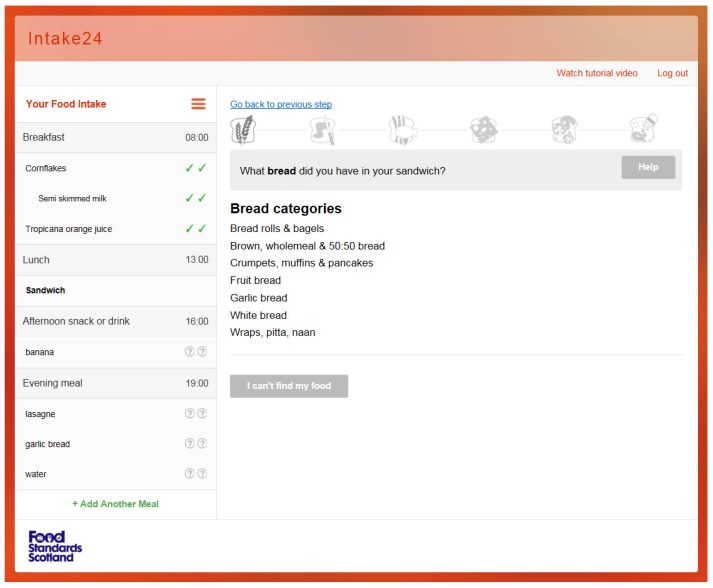
INTAKE 24 sandwich wizard. First stage; what bread did you have? Each of the greyed out icons represent the stages in the sandwich wizard.

The system recognises the term ‘sandwich’ and synonyms, such as ‘roll’, ‘butty’, or ‘wrap’. The system returns ‘Build my sandwich; at the top of the food list and selecting it initiates the Sandwich Wizard; see Figure 3. The user is asked a series of questions regarding the components of the sandwich, as follows:

1. What kind of bread did you have in your sandwich?
2. What kind of spread did you have in your sandwich?
3. What kind of meat or fish did you have in your sandwich?
4. What kind of cheese or dairy product did you have in your sandwich?
5. What kind of extra filling did you have in your sandwich?
6. What kind of sauce or dressing did you have in your sandwich?

For all the questions above there are relevant categories listed below from which the user can select the exact food. For questions 2–6 they have the option to say ‘I didn’t have any’. The user is asked question 5 repeatedly to ensure all components of the sandwich are captured, until they select ‘I did not have any other fillings’. The user is asked the final question regarding sauces and then is taken through the portion estimation stage.

To make it easier for the user, some brand names are included in the database, e.g., Warburtons seeded batch and Hovis 50/50. 

This feature was further adapted for salads. The salad wizard works in the same way. The system recognises that ‘salad’ has been entered by the user and asks ‘What items did you have in your salad?’. The user can select as many foods as they consumed and then click ‘I did not have any more’. The user is finally asked ‘Did you have any sauces or dressings on your salad?’. The user is then guided through the portion estimation stage for each salad item entered. 

## 5. Portion Size Estimation and Additional Foods

**Figure 4:**
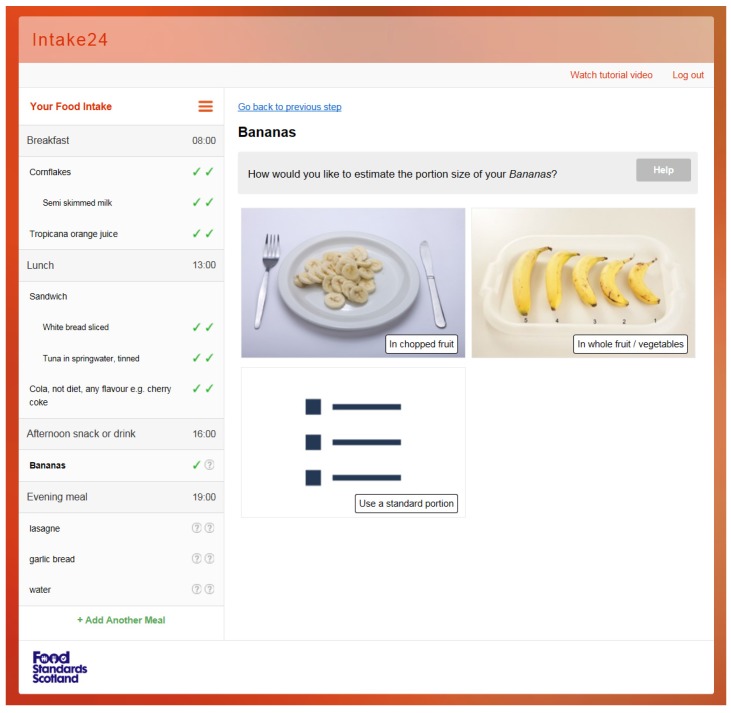
Portion size estimation in INTAKE24. Banana: chopped (as served), whole fruit (guide image), and standard portion.

The portion size estimation stage is part of the second pass of the recall. Once they have added all their foods and drinks, they are directed to select the closest match in terms of food type in the food database list and then to select the portion size for each food and drink entered. The original SCRAN24 system included over 2000 photographs of over 100 foods for portion size estimation from the validated Young Person’s Food Atlas (YPFA) [16]. There are four different ways to estimate portion size in INTAKE24; two different types of images (as served/guide) and sliding scales for drinks and standard portion descriptions. An example of the choice of method of portion estimation is shown in Figure 4. Guide photographs display a range of items, which are available in pre-determined amounts, e.g., slices of bread or biscuits. The number of items in the guide photos is dependent on the food and the range of different sizes/portions available. For example, apples have a range of four different sizes to choose from, and for sweets there are 23 items from which to choose in one guide photo.

For the ‘as served’ images, the portion sizes depicted are based on the amount of foods served to children taking part in the National Diet and Nutrition Survey [12]. The range is from the 5th to 95th centile of the weight of food served for participants aged 11–16 years. Up to seven portion images are depicted. The range of portion sizes in the system have been checked against the range of portion sizes reported in the most recent National Diet and Nutrition Survey [17] of adults which used weighed food diaries, and the portion sizes cover the range consumed by adults in all but a few cases.

Although the YPFA has previously been tested and validated, large discrepancies in estimated intakes were still found for a small number of foods. As part of the development of INTAKE24 we investigated whether different methods of presentation of the foods in the food photographs could improve the accuracy of portion size estimations. For example, for butter/margarine the range of photographs were extended from butter on bread only to also include images of portions of butter on a bread roll, a scone and also on a knife. Sixty participants were recruited from a local secondary school and Newcastle University; 30 were aged 11–17 years and 30 aged 18–24 years. All foods were prepared and presented to the participants as they would be served if they were to be consumed, i.e., in a bowl, on a plate, in a mug, in a glass. The weights of the foods and drinks were recorded; these were chosen to represent a range of the portions usually consumed and did not exactly match any of the portion photographs (with the exception of the packaged items). The participants were asked to estimate the portion size using the original YPFA photos and the new photos in a randomised order. If the new images proved more accurate these were included in the system, (*n* = 15) [18]. 

In total, over 400 new foods and 700 new photographs were added to INTAKE24. These include regional foods (Scotland), alcoholic drinks, and common foods that were missing from the system. These were compiled from searches of supermarket websites and included foods such as chorizo, roast duck, soya alternatives to dairy products, and a larger range of breakfast cereals.

To make searching for foods easier, some of the foods and food categories were re-named to be more user-friendly. For example, ‘Short sweet biscuits, e.g., Shortcake biscuit, Lincoln’ was re-named ‘Shortcake biscuit’. For some foods, however, examples were added to the food description; for example, ‘Dry cider’ and ‘Sweet/medium cider’ were re-named ‘Dry cider e.g., Strongbow’ and ‘Sweet/medium cider e.g., Woodpecker’. 

The food codes in INTAKE24 are linked to the most comprehensive up to date version (year 4) of the Nutrien Databank from Public Health England. Whole dishes are also included, such as pasta dishes, casseroles, curries, etc. Within the food database, the foods are also assigned to a food group, or, in the case of composite dishes, to multiple food groups to allow data output in terms of food groups as well as nutrients.

Two researchers worked independently assigning codes to all the foods contained within the system. A systematic procedure was in place to discuss the codes and categories assigned in a feedback loop with the remaining research team to ensure reliability. Matches (same databank code assigned to the same food) and discrepancies (different codes assigned to the same food) were checked at regular intervals throughout the coding process. Discrepancies were resolved through extended discussions with the research team and included a steering group member from Public Health England who also checked the re-coding to ensure consistency with National Diet and Nutrition Survey coding, while advising on the most appropriate codes to assign.

## 6. Small Scale Comparison of INTAKE24 Against Interviewer-Led Recalls

The participants completed an interviewer-led recall immediately after having completed INTAKE24. The interviews were always conducted in this order as the main purpose of the interviewer-led recall was to identify any foods or drinks which were not reported in INTAKE24 but which the interviewer could elicit. The interviewer-led recalls were coded with the same databank food codes used in INTAKE24. Although the numbers completing the recalls were small, we also conducted a comparison of food intake by INTAKE24 with the interviewer-led recall to give an indication of the agreement between the two methods. A ratio for each individual’s total intake of kJ and key nutrients was calculated, using the formula below:

Ratio = estimated intake (INTAKE24)/estimated intake (interviewer-led) [19].

Ratios of less than 1 indicate an under-estimation of intakes using INTAKE24 and above 1 represent an over-estimation, compared with the interviewer led recall. Table 2 illustrates the changes in agreement between the two methods through the four rounds of testing. The closer the value is to 1, the better the agreement. For all nutrients (except for vitamin C), the agreement was improved using INTAKE24 compared with SCRAN24. Agreement also improved in each round of testing, from the INTAKE24 prototype tested in Round 2 to the final version in Round 4, despite the decrease in completion time.

A further relative validation has been conducted on a refined version of INTAKE24 and is reported elsewhere [20]. 

## 7. Usability Scores

The System Usability Scale (SUS) is regarded as being a reliable and robust method of evaluation for digital technologies, while taking into consideration the context of their use. The SUS score is calculated using a specific formula for each of ten statements based on the Likert scale result. Statements included ‘I think I would like to use this system frequently’ and ‘I feel very confident using this system’ [13]. Scores can range between 0–100, and having a score above 80 is considered excellent [21]. The SUS scores for INTAKE24 throughout the four stages of testing are shown in Table 3. The scores for each stage of testing were above average, and overall INTAKE24 can be considered user friendly with a score of 83/100 for the final system tested. This compares favourably with other UK online dietary assessment tools like myfood24, which stated a usability score of 74/100 [22]. We performed a Kruskal Wallis test on SUS scores from all participants at each of the stages of testing, and the results indicate a significant difference with a *p* value 0.002.

## 8. Additional Features

### Welcome Text, Contextual Questions, and Final Review

**Figure 5:**
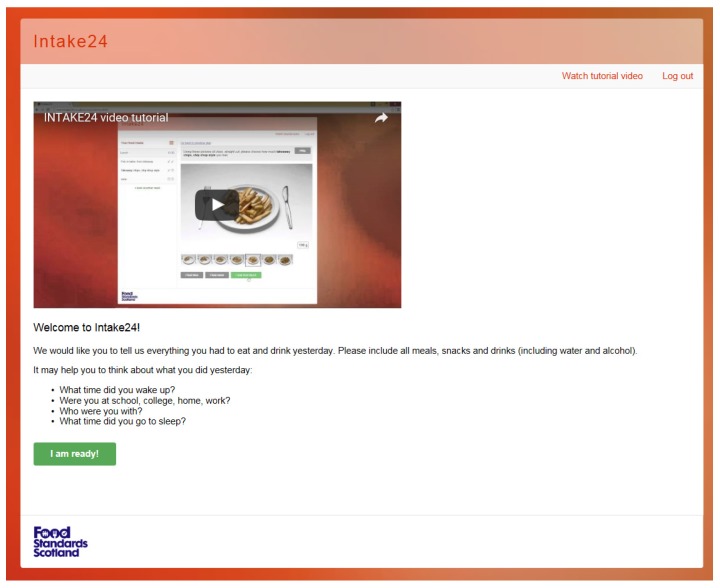
Welcome screen INTAKE24, video tutorial, and modifiable welcome text.

**Figure 6:**
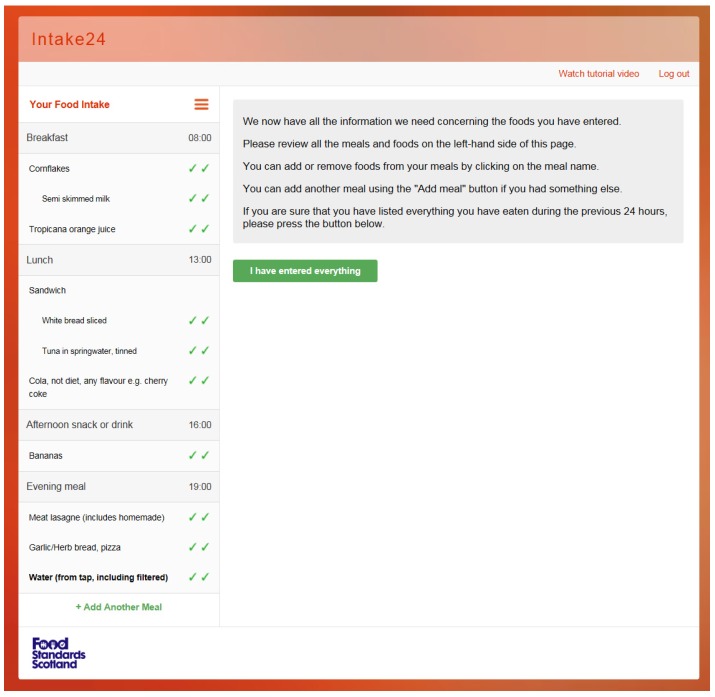
Final review stage in INTAKE24.

The welcome screen of the survey helps to contextualise the recall with questions to help the user to think about their previous day’s intake; see Figure 5. Users are requested to enter all foods and drinks consumed from waking-up through until bedtime. The text is easily modifiable and can be changed depending on the participants taking part in a survey. In addition to the recall, there is scope to add in questions around dietary behaviours before the final review; for example, ‘Where did you buy the food?’, ‘Did you eat this alone?’, or ‘Did you eat this in front of the TV?’ These questions can be customised, depending on a study’s specific research questions. The final review summarises the dietary recall and allows the user to make any final changes before submitting the survey; see Figure 6. 

## 9. Researcher Interface

There is a separate interface for researchers running a dietary survey. Researchers can log onto the INTAKE24 system using a staff username and password. This directs them to the researcher interface, where they are able to carry out five main functions:

Start, suspend, or end a survey.

Download the survey data as a .csv file for easy upload to statistical packages.

Download an activity report. This informs the researcher of the number of recall submissions; the mean, minimum, and maximum completion times; and the submission dates for each participant.

Upload usernames and passwords for participants.

Update the survey schedule (start and end dates).

## 10. Summary

The evaluation focused mainly on the usability of the system (e.g., how easy it is to learn and use) and on the UX while interacting with the system (e.g., how satisfying, enjoyable, and motivating the system is to use) [9]. Integrating observation and post-completion interviews allowed us to maximise the information obtained to feed into the design process, amending and improving the system to maximise usability, as well as increasing the accuracy of the dietary recall. 

Although only 80 participants were used in the development phase, the results comparing INTAKE24 with an interviewer-led recall were promising, as agreement improved in each round of user testing (Round 2 to Round 4). Involving end users in the design and development has made invaluable contributions to the overall system and resulted in a system with an excellent SUS score. Further work has carried out a relative validation of INTAKE24 against interviewer-led recall in 168 people reported in a separate paper [20].

## 11. Future Work

The system is kept up to date by adding to the database any missing food and drink items that have been identified by current users of the system. The use of INTAKE24 among an older adult population is underway. Further improvements since development include a video tutorial, a recipe editor, and specific help clips. In addition, the system is being translated into other languages for international use such as Arabic, Portuguese, and Danish. This process involves studying the food culture in these countries with external collaborators. In doing so, we re-appropriate the system to be used in a different context, not just through translation of language but also by adding culturally relevant foods and food composition data. Ultimately, our future aim is to validate the system with recovery biomarkers. 

A demo of the system can be found at https://INTAKE24.co.uk/.

## Figures and Tables

**Table 1:** Summary of modifications throughout system development, after user testing.

Round 1	Round 2	Round 3
‘Flat’ interface introduced, i.e., all tasks performed on same screen.	Separate text entry box for food and drink (instead of all together)	Added Frequently Asked Questions
Aesthetics of system; colour scheme/layout	Instructions completed for each stage of the multiple pass	Welcome screen text and final review text
	Sandwich and Salad wizard implementation	Added task specific help messages
	Adding associated prompts	

**Table 2:** Agreement between INTAKE24 and interviewer-led recalls at all four stages of testing.

Ratio INTAKE24: Interviewer-Led	User Testing			
	Round 1 (SD) (SCRAN24)	Round 2 (SD)	Round 3 (SD)	Round 4 (SD)
Weight of food (g)	0.90 (2.48)	0.73 (1.24)	0.87 (1.30)	1.05 (1.31)
Energy (kJ)	0.84 (1.69)	0.80 (1.28)	0.84 (1.30)	0.89 (1.24)
Carbohydrate (g)	0.92 (1.95)	0.88 (1.48)	0.85 (1.35)	0.94 (1.30)
Protein (g)	0.80 (2.57)	0.77 (1.40)	0.86 (1.31)	0.96 (1.27
Fat (g)	0.78 (1.63)	0.52 (1.94)	0.81 (1.37)	0.81 (1.30)
Vitamin C (mg)	1.09 (6.36)	1.15 (2.17)	0.89 (2.44)	1.22 (2.26)
Iron (mg)	0.81 (2.15)	0.78 (1.68)	0.84 (1.47)	1.03 (1.37)

SD: standard deviation.

**Table 3:** SUS (System Usability Scale) score for INTAKE24 from all four stages of testing. * *p* < 0.05 = statistically significant.

	INTAKE24 SUS Score				
	Round 1 (SCRAN24)	Round 2	Round 3	Round 4	*p* Value *
**SUS Score**	72	71	82	83	0.002
